# Conventional Laparoscopy Is the Better Option for Tubal Sterilization Reversal: A Closer Look at Tubal Reanastomosis

**DOI:** 10.1089/whr.2021.0039

**Published:** 2021-09-03

**Authors:** Anita Madison, Lamia Alamri, Adina Schwartz, Marja Brolinson, Alan DeCherney

**Affiliations:** ^1^ The Department of Obstetrics and Gynecology, Woman's Hospital Louisiana State University School of Medicine, Baton Rouge, Louisiana, USA.; ^2^ The Department of Obstetrics and Gynecology, Vanderbilt University Medical Center, Nashville, Tennessee, USA.; ^3^ Program in Reproductive Endocrinology and Gynecology, Eunice Kennedy Shriver National Institute of Child Health and Human Development, National Institutes of Health, Bethesda, Maryland, USA.

**Keywords:** fimbriectomy, IVF, neosalpingostomy, permanent sterilization, tubal ligation

## Abstract

***Background:*** Permanent sterilization is one of the most common methods of birth control in the United States and around the world. A small subset of women will regret their decision and desire future fertility. For these women, the options include *in vitro* fertilization (IVF) or surgical reversal. Surgical reversal, specifically *via* tubal reanastamosis, is an important choice to consider. Surgical reversal can be accomplished *via* three different general approaches including laparotomy, conventional laparoscopy, and robot-assisted approaches. Unfortunately, surgical reversal is becoming a lost art.

***Objective:*** To compare and contrast pregnancy success rates, ectopic pregnancy rates, and cost between the surgical methods and IVF.

***Methods:*** We conducted a literature review via Pubmed with keywords as listed below.

***Conclusion:*** Laparoscopic tubal reanastomosis is the best approach for women <40 years of age due to pregnancy outcomes that are comparable to other methods, cost effectiveness, and favorable safety profile of minimally invasive surgery.

## Background

Permanent sterilization continues to be a popular form of birth control in the United States. Data from the national survey of family growth show that female sterilization is the most common method of contraception.^1,2^ In the United States, ∼21.6% of women aged 30–39 years and 39.4% of women aged 40–49 years rely on some form of permanent sterilization.^1^ In 2019, the overall global prevalence of permanent sterilization was 24% in women aged 15–49 years.^3^ There are some global variations in prevalence, with the highest being in central and southern Asian countries as well as in Latin American and Caribbean countries, 21.8% and 16%, respectively.^3^ Regret remains a significant risk however, especially in certain groups including age <30 years, low parity, low socioeconomic status, and performance of the procedure in the postpartum period.^4^ In the United States, 30% of women will regret their decision and ∼1% will seek reversal.^5^ For the subset of women interested in future fertility after this procedure, the options include surgical reversal or *in vitro* fertilization (IVF). Many different factors must be considered when counseling patients on these two options including but not limited to age, ovarian reserve, cost, religious beliefs, surgeon skill level, and other processes affecting fertility.^6^

The different methods of permanent sterilization include the use of silicone rubber bands, clips, and partial salpingectomy including fimbriectomy and coagulation, all of which are amenable to reversal. Table 1 demonstrates the percentages of different methods of sterilization procedures. The success rates of reversal depending on the method of sterilization used are mixed. Some studies found a statistically significant increase in pregnancy rates after reversal of Filshie clips and Falope rings, whereas other studies did not.^7–9^ Furthermore, when patients undergo a Kroener operation, more commonly known as a fimbriectomy, it becomes impossible to perform a reanastomosis. This results in the need to perform a neosalpingostomy/fimbrioplasty with the hope that the oocyte can be collected by the ampullary portion of the tube. Additional factors that may influence the decision to proceed with operative management take into account the amount of tube remaining, location of the anastomosis, and time elapsed since sterilization.^10^ As such, review of prior operative reports becomes a vital part of the preoperative assessment.

**Table 1.** Common Sterilization Procedures in the United States

Method	Percentage^a,35^
Bipolar coagulation	21
Unipolar coagulation	13
Silicone rubber band	31
Spring clip	15
Interval partial salpingectomy	4
Postpartum partial salpingectomy	15

Percentages of different sterilization procedures that were performed in 10,685 women enrolled in the U.S. collaborative review of sterilization.

^a^ Sum of percentages equal to 99% due to approximation.

Although there has been a shift toward IVF in recent years, surgical tubal reversal remains a viable and important option for a select patient population. Tubal anastomosis is becoming a lost art that many reproductive endocrinologists have unfortunately abandoned. This review describes why traditional laparoscopy is a superior approach, as we summarize the chronological developments in surgical tubal reversal techniques, including a look at different success rates and a comparison with IVF. In addition, we report a case of neosalpingostomy after fimbriectomy for permanent sterilization reversal.

## Laparotomy

The first procedure performed for tubal reanastomosis (TA) was done in the 1970s *via* laparotomy.^11^ This technique involved creating an abdominal incision to access the Fallopian tubes. Excision of the occluded ends was performed, and methylene blue or indigo carmine dye was injected into the uterus as well as the fimbriated end to confirm patency before end-to-end anastomosis. This was frequently followed by placement of a splint that was removed weeks later to prevent reocclusion.^11–13^ In the late 1970s, the microscopic camera was introduced in open surgery to allow for higher precision.

The introduction of the microscope brought in the ability to perform a two-layer technique wherein the end-to-end anastomosis consisted of suturing the muscularis and serosal layers separately.^12^ The technique involved placement of a catheter into the proximal end of the tube and then into the distal end. Clips were used to hold the tubal segments in place to assist in suturing each layer.^12,14^

During the infancy of TA, pregnancy success rates *via* laparotomy depended mainly on patient age, reanastomosis technique, and sterilization type and varied between 40% and 80% as reported by several authors.^11,13,15^ It has been established that one of the most important factors for success is a tubal length of ≥4 cm.^13,15^

## Conventional Laparoscopy

Laparoscopic surgery for tubal anastomosis began in the late 1980s and has been improving ever since with advancing technology.^16,17^ Similar to the open microsurgical approach, the two-layer technique is usually employed. The procedure involves microsuturing using 6–0 to 10–0 sutures. Another alternative has been the replacement of traditional sutures with barbed sutures, allowing for a simpler technique that does not require laparoscopic knot tying.^18^ Tubal patency is confirmed both intraoperatively and with subsequent hysterosalpingogram.

Comparison studies revealed the success rate to be similar between laparotomy and laparoscopic reanastomosis with an overall pregnancy rate of 80% and intrauterine pregnancy rates of 77%–78% *via* either approach.^17,19^ Time from surgery to pregnancy is also comparable between laparotomy and laparoscopy. When comparing the two techniques, operating time for laparoscopy is longer than that for laparotomy. In addition, it is well established that laparoscopy is associated with a longer operator learning curve. However, the mean hospital stay after laparoscopy is shorter postoperative discomfort and analgesic requirement is reduced and cosmesis is superior compared with laparotomy.^17,19,20^

## Robot-Assisted Laparoscopy

The first case of robotic surgery in gynecology was a tubal anastomosis in 1999 reported by Falcone et al.^21^ Although laparoscopy provides a minimally invasive approach, it has some limitations in accomplishing TA. Some of the drawbacks associated with laparoscopy include a long learning curve, accentuation of physiological tremor, image instability, fixed instrument axis, and overall high difficulty level with intracorporeal suturing.^22^ Using the robot, the surgeon has the advantage of utilizing three-dimensional imaging, a magnified operative field, tremor filtration technology, enhanced dexterity, and ergonomics.^22^ Given that TA requires high precision and atraumatic suture placement, robotic surgery became a very attractive method.

Analogous to microsurgical open procedures and traditional laparoscopy, the surgical technique employed involves resection of the occluded tubal segments followed by end-to-end anastomosis of the distal and proximal segments *via* the use of small diameter monofilament suture (≤5 mm).^22^ Tubal patency is then assessed with both chromopertubation at the time of surgery and hysterosalpingogram postoperatively.

In a retrospective cohort study by Caillet et al., a pregnancy rate of 71% and live birth rate of 62% were observed in 97 women undergoing robotic TA.^23^ These results were comparable with what has been previously reported in the literature.

A robotic approach is not without disadvantages. One of the biggest limiting factors in using the robot is cost. Robots are expensive with prices ranging between $1 million to $2.5 million per unit, and often require high maintenance fees.^24^ A meta-analysis including 27 randomized control trials comparing treatment outcomes between conventional laparoscopy and robot-assisted techniques across different surgical subspecialties and procedures found that robotic surgery had significantly increased operative times, intraoperative complications, and a trend toward increased conversion to laparotomy.^25^ The loss of tactile feedback has been a controversial issue. It is reasonable to assume that loss of feedback can cause unnecessary tissue damage or inadequate tension on knots, for example, but this has been debated, especially in the setting of microsurgery.

## *In Vitro* Fertilization

Interest in tubal surgery has waned mainly due to the advantages of assisted reproductive technology (ART), specifically IVF, which has an overall live birth rate of 28.5%–35% per cycle, faster time to pregnancy, and smaller rate of complications.^26,27^ IVF involves stimulation of the ovaries to produce a large number of ovarian follicles through the use of gonadotropins with the ultimate goal of retrieving mature follicles for freezing or embryo creation. This effectively allows us to bypass fallopian tube pathology that may hinder or prevent pregnancy.

It is difficult to extrapolate a direct comparison between surgical success rates versus IVF success rates since surgical success is reported as pregnancy per person, whereas IVF success is mostly reported as pregnancy per cycle; this does not allow for an accurate head-to-head comparison. However, two studies have compared pregnancy outcomes between surgical reanastomosis and IVF on a per patient basis. Boeckxstaens et al. studied pregnancy outcomes between surgical reanastomosis and IVF and found a 59.5% liveborn delivery rate in the TA group compared with the 52% liveborn delivery rate in the IVF group non significant (NS).^28^ They also found that age was the only factor that significantly influenced delivery rates. The cumulative delivery rate for patients aged <37 years was 52.4% after IVF and 72.2% after reversal (*p* = 0.012), whereas cumulative delivery rates for patients aged 37 years or older were 51.4% and 36.6% (NS).^28^ The time from first treatment to delivery was 21 months compared with 14 months in the IVF group. In addition, the ectopic pregnancy rate was 3.6% in the reanastomosis group compared with 0% in the IVF group.^28^ Longer time to pregnancy may discourage many patients from attempting a TA.

In 2009, Malizia et al. sought to estimate the cumulative live birth rate in 6164 women after six IVF cycles. Overall, using conservative estimates, the cumulative live birth rates after six IVF cycles was 51% per patient. However, when stratifying by age, the rate was 65% for women under 35 years of age. The corresponding rate for women aged 40 years or greater was 23%.^29^

With regard to tubal ectopic pregnancies, Schippert et al. found a nonsignificant increase in tubal ectopic pregnancies between reanastomosis and ART (6.7% vs. 5.6%, NS). In this study, the pregnancy rate was 73%. However, the study does not describe the types of anastomoses or length of Fallopian tubes. The patients' ages ranged from 26 to 42 years with a median age of 35.4 years.^30^

Messinger et al. performed an analysis of cost effectiveness comparing IVF with TA and found that TA was more cost effective for women <40 years. For TA, the average costs per ongoing pregnancy (pregnancy >20 weeks assuming live birth) was $16,315, $23,914, and $218,742 for <35-, 35-to 40-, and >40-year-old women, respectively. For women who underwent IVF, the cost was 32,814, $45,839, and $111,445, respectively. This study performed a robust cost analysis that included market price charges for a TA, single cycle fresh IVF, and single cycle frozen IVF transfers. These charges included everything from the physician visit to the procedure and hospitalization costs. They also included the cost of managing an ectopic pregnancy, spontaneous abortion, or IVF complication such as ovarian hyperstimulation syndrome.^5^ Cost constraints are more pronounced in low-resource countries, where IVF is unavailable or inaccessible to a large portion of the population.^31^ Surgical intervention in this setting becomes a practical and cost-effective method.

## Discussion

When comparing approaches, laparoscopy seems to be the best approach for women <40 years of age due to pregnancy outcomes similar to other methods, overall cost effectiveness, and the favorable safety profile of minimally invasive procedures. A meta-analysis done in 2017 by van Seeters et al. investigated fertility outcomes of different surgical methods for reversal of female sterilization as compared with IVF.^32^ The pregnancy rates were comparable among the three surgical approaches. Microsurgery *via* laparotomy had a pooled pregnancy rate of 68% with an ectopic rate of 10.4%, laparoscopic tubal reversal had a pooled pregnancy rate of 65% with an ectopic rate of 5.6%, and robotic tubal reversal had a pooled pregnancy rate of 65%. Unfortunately, there were only two robotic studies that quoted ectopic rates, which had a pooled result of 15%. Here you can see that pregnancy outcomes were similar with a relatively lower ectopic rate for laparoscopy. When comparing laparoscopic tubal reversal with IVF, the pregnancy and live birth rates tend to be higher in the laparoscopy group.^5,32,33^ Table 2 demonstrates the findings of the mentioned study, further illustrating the comparable outcomes between the methods.

**Table 2.** Pregnancy Rate and Ectopic Pregnancy Rate (%) by Method

Method	Pregnancy/live birth rates (%)	Ectopic pregnancy rate (%)
Laparotomy (microsurgery)^32^	68^a^ (95% CI: 58–71)	10.4^a^
Laparoscopy^32^	65^a^ (95% CI: 61–74)	5.6^a^
Robotic^32^	65^a^ (95% CI: 59–72)	15^a^
*In vitro* fertilization^28,29,34^	51^b^	0

^a^ Pooled pregnancy rates with varying time intervals from surgery (6 months to 6 years).

^b^ Conservative estimate for cumulative live birth rates after six cycles.

Laparoscopy is also the overall most cost-effective approach. When compared with laparotomy, it is almost $500 less per operation due to shorter hospital stay. When compared with robotic surgery, it is significantly less expensive as already discussed. IVF tends to be less cost effective as age decreases. Studies have shown that for women under age 40 years, tubal reversal is the more cost-effective option by thousands of dollars.^5,32^ Based on review of the literature, laparoscopy is the better option in women <40 years old. Conventional laparoscopy is associated with shorter hospital stays, excellent cosmesis, and less analgesic requirements compared with laparotomy. In addition, when compared with robot-assisted surgery, laparoscopy has decreased intraoperative time, cost, and complications.^25^ Finally, in comparison with ART, benefits of TA surgery include spontaneous conception, recurrent opportunities to conceive without the need for additional treatment or procedure, as well as decreased cost (Table 3).^27^ In contrast, IVF tends to be the better option for women 40 years and older due to the lower success rates of tubal reversal in this age group.

**Table 3.** Advantages of Laparoscopic Tubal Reanastomosis

One time minimally invasive procedure
Ability to attempt conception every month and conceive more than once without addition procedures
Better time to recovery, cosmesis, shorter hospital stay, and lower analgesic requirement than laparotomy
Decreased cost compared with IVF and robotic assisted
Avoid IVF risk such as multiple gestations and OHSS

Practice committee of the ASRM role of tubal surgery in the era of ART: a committee opinion. FertilSteril.

ART, assisted reproductive technology; IVF, *in vitro* fertilization; OHSS, ovarian hyperstimulation syndrome.

The fact that extended surgical training is required to become proficient in this procedure should not be discouraging. On the contrary, it should generate a new sense of enthusiasm and commitment to refine surgical skills. The reproductive endocrinologist should hang on to and seek opportunities to perform tubal reversal in the appropriate clinical setting. After all, one cannot always take the easy way out.

## Abbreviations Used

ART: assisted reproductive technology
IVF: *in vitro* fertilization
NS: non significant
TA: tubal reanastomosis
